# Carabid Beetles (Coleoptera) as Indicators of Sustainability in Agroecosystems: A Systematic Review

**DOI:** 10.3390/su15053936

**Published:** 2023-02-21

**Authors:** Maria M. Makwela, Rob Slotow, Thinandavha C. Munyai

**Affiliations:** 1 School of Life Sciences, College of Agriculture, Engineering and Science, University of KwaZulu-Natal, Private Bag X01, Scottsville 3209, South Africa; 2 Oppenheimer Fellow in Functional Biodiversity, Centre for Functional Biodiversity, School of Life Sciences, College of Agriculture, Engineering and Science, University of KwaZulu-Natal, Private Bag X01, Scottsville 3209, South Africa

**Keywords:** agricultural management type, biodiversity, ground beetles, ecological monitoring, functional diversity

## Abstract

The sustainability of agroecosystems is at risk owing to continuous anthropogenic disturbance. As such, there is a need to evaluate indicator taxa that may be used to monitor the health of agricultural management systems. Carabid beetles are ubiquitous and functionally crucial in agroecosystems while at the same time are sensitive to the changes caused by management practices. Their quick response to anthropogenic disturbances has been proposed as a practical and realistic tool for monitoring the sustainability of agricultural practices. However, there is still disagreement about carabids as possible indicators of agroecosystem sustainability. We conducted a systematic review of the responses of carabid beetles to agricultural systems in different biogeographical areas. We examined whether these beetles could serve as potential indicators of agroecosystem sustainability. The ISI Web of Science, Google Scholar, and Scopus were used to search for papers published from 2000–2019. In total, we included 69 studies indicating the use of carabids to monitor the impact of management practices in agroecosystems. Most studies were conducted in European countries (n = 37), while Southern Africa and East Asia countries were significantly under-represented (n = 10). Carabid beetle response to agroecosystems varied between management practices, with biodiversity indices (n = 41: positive 60%, negative 19%, and neutral 19%) being the most measured response variable, followed by functional diversity (n = 28: positive 67%, negative 25%, and neutral 7%). Overall, our findings highlight the need for more research in underdeveloped countries, to investigate the potential of overlooked carabids and include response variables measuring functional diversity in assessing the sustainability of agricultural management. This will assist policy makers and land managers in making active and informed decisions about agroecological disturbances and management.

## 1 Introduction

Agricultural intensification is one of the main causes of the biodiversity crisis [1], with repercussions for the functioning and sustainability of agroecosystems [2,3]. Agricultural management systems that involve the continuous use of pesticides result in habitat degradation, the conversion of semi-natural habitats to cropland, and the dominance of a few plant species in ever larger areas [4–6]. These significant challenges impact the ecological processes that provide the functions necessary for sustainable production [7]. Moreover, management practices are confronted with numerous challenges on multiple fronts, ranging from meeting the food demand for the growing population to dealing with climate change effects [8,9]. Concerns about the negative effects of high-input, industrially managed agriculture have prompted a call for sustainable management practices [10].

The implementation of sustainable agricultural practices should include cost-effective monitoring techniques that can be used to detect environmental changes, assess management performance, and provide warning signals for imminent ecological transitions [11]. Indicators are species or groups of species that are easily monitored and whose status reflects or predicts the condition(s) of the environment where they are found [12]. Indicator species may be a helpful tool for addressing agricultural intensification difficulties [13]. For decades, changes in key indicator species have been used to underpin increasing concern about the necessity of biodiversity conservation and sustainability in the face of accelerated environmental change induced by human activities. Subsequently, the use of indicators has been extended to aquatic and terrestrial invertebrates used to detect environmental impacts in freshwater [14–16] and different ecosystems [12,17–23]. Despite the extensive history of prospective indicator species, surprisingly, few studies have been conducted on indicators for sustainable agroecosystems [24,25]. Therefore, using taxonomic or functional groups that are sensitive to ecological change in agroecosystems might be a helpful tool to monitor the resilience and health of management practices.

Among edaphic arthropods, carabid beetles are regarded as excellent indicator species due to their abundance and diversity, well-known taxonomy, ease of sampling, and costeffectiveness [12,26]. Carabids are particularly suitable for examining subtle effects of agroecosystem management practices such as pesticide use, depth of tillage, soil quality, moisture, and landscape heterogeneity, because certain species are stenotopic and thus intrinsically sensitive to environmental conditions [18,27–29]. Other biodiversity assessments have reported carabid responses to grassland management practices [30–33]. The importance of carabids in agroecosystems is critical due to their economic and functional value, acting as natural enemies of pests or components of trophic chains that support biodiversity [34,35]. Though carabids have been well studied taxonomically and ecologically in agroecosystems worldwide [36], most studies in Africa have documented the carabid beetle diversity in savanna biomes [37], forest–grassland mosaics [38], vineyards [39], and few in cereal agroecosystems [30,40]. Notably, their use as indicators of agroecosystem sustainability remains a challenge due to a lack of data on their ecological response, particularly under different agricultural management scenarios [21,36,41–44]. A detailed knowledge of the response of carabids in various management practices can provide insight into the health of agroecosystems. This review aims to provide a global and comprehensive overview of the use of carabid beetles in agroecosystems and assesses their inclusion as sustainability indicators. The focus is on conservation and biodiversity studies, with a geographical emphasis on agroecosystems where most research has been conducted, namely across semi-natural grasslands and field crops.

## 2 Materials and Methods

**Figure 1:**
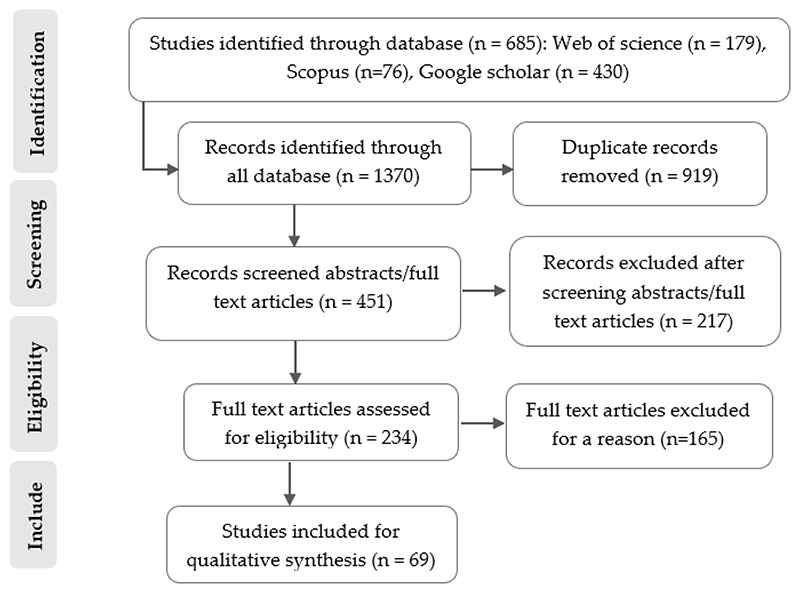
A flow diagram showing the systematic review process (adapted from PRISMA guidelines).

To summarize the ecological response of carabid beetles to agricultural management practices in different geographical regions, we conducted a systematic review using the Preferred Reporting Items for Systematic Reviews (PRISMA) statement [45], and checklists [46], (Figure 1, see Supplementary Information PRISMA checklists 2020—Tables S2 and S3). PRISMA is a standard protocol for conducting objective and reproducible systematic reviews to improve scientific transparency. We selected studies that examined cropland and semi-natural grassland because these agroecosystems are managed differently (from semi-natural habitats to homogeneous monocultures, crop rotations, pastures, organic farming, and diversified and conventional tillage), which affects carabid beetle biodiversity and functionality (Table 1, see Supplementary Information—Table S1).

### 2.1 Search and Selection of Publications

We searched the ISI Web of Science Core Collection (http://www.isiwebofknowledge.com), Scopus (https://www.scopus.com), and Google Scholar (https://scholar.google.ca) for peer-reviewed publications published on all continents. We used the following search term to find studies on the effects of agroecosystem management practices on carabid beetles: (“agri-environmental programmes” OR “organic farming” OR “sustainable farming” OR “diversified farming” OR “integrated farming” OR “conservation farming” OR “conventional tillage” OR “intensive farming” OR “semi-natural grassland AND *Beetles”, AND *Carabidae”, AND “ground beetles” AND *Indicator”). To find the response variables, we used the following search term: (“abundance” OR “richness” OR “diversity” OR “composition” OR “functional diversity” OR “weed predator” OR “generalist” OR “specialist” OR “morphological traits” OR “trophic guilds”). All journal citations and abstracts were imported into the Mendeley online importer reference management software (https://www.mendeley.com) and their titles, keywords, and abstracts were checked.

### 2.2 Data Extraction and Synthesis

We focused on extracting data on language (English), year of publication (March 2000 to April 2019), biogeographic region (country/continents), agricultural management type (conventional tillage, organic farming, diversified farming, conservation agriculture, and grazing), diversity (richness, evenness, composition, abundance, and active density), functional traits (body size and dispersal ability), and trophic guilds (predators, granivores, and omnivores) (Table 1). We also evaluated each publication according to whether the authors reported negative, positive, or neutral effects of different agroecosystem management practices on carabid beetles. Grey literature, books, conference proceedings, technical reports, and unpublished data were not considered in the review conducted here.

## 3 Results

### 3.1 General Overview

The initial search for relevant articles resulted in 1370 articles. After eliminating duplicates (919 articles), the search criteria yielded 451 articles across all databases. The titles, abstracts, and keywords of 451 articles were screened, with 217 excluded. Critical appraisal of the 234 studies that met the relevance criteria led to the exclusion of 165 studies due to low or unclear validity. Consequently, 69 studies showing the response of carabid beetles to different management practices in agroecosystems were used for the qualitative synthesis. The search, screening, and inclusion of studies are schematically summarized in the flow chart below (Figure 1).

### 3.2 Trends in Carabid Beetle Studies and Status in Agricultural Management Systems

**Figure 2:**
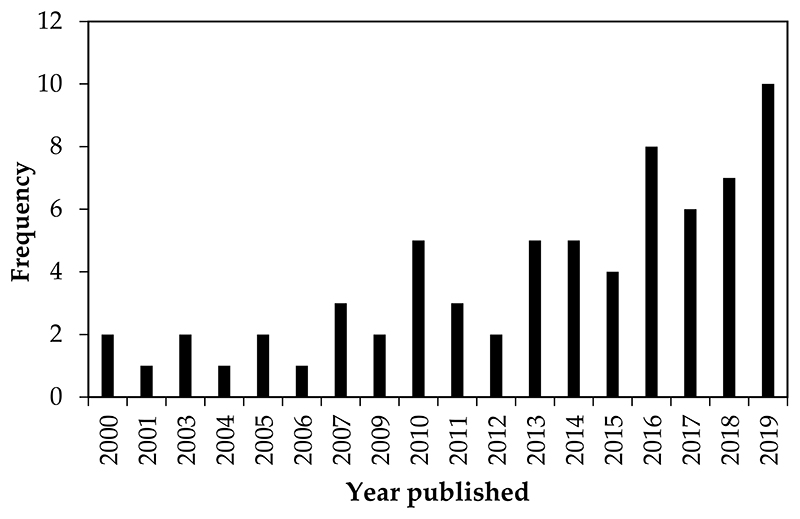
Publications (n = the total of 69 screened articles) that investigated the response of carabid beetles to agricultural management practices between 2000 and 2019.

**Figure 3:**
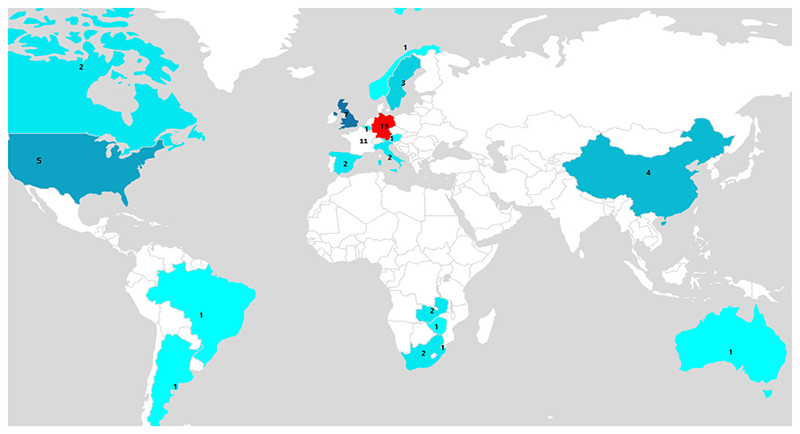
Geographic locations of the 69 included peer-reviewed studies on the word map.

**Figure 4:**
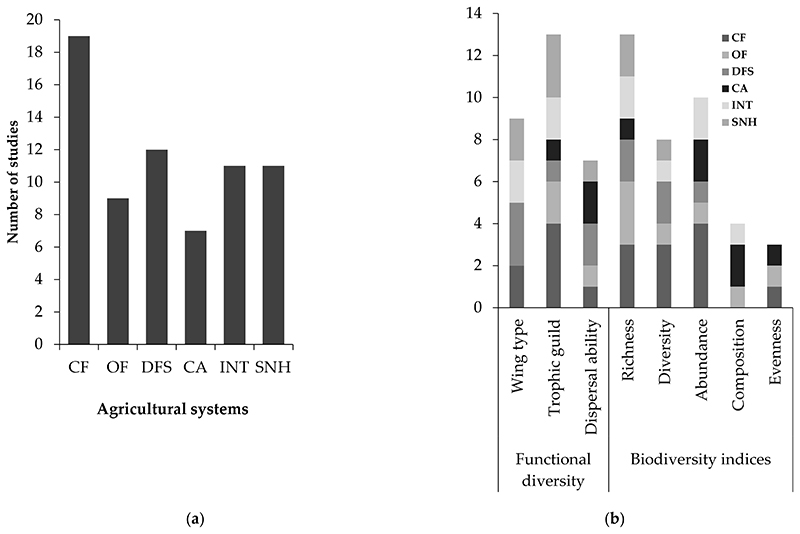
Summary of number of papers published on (**a**) agricultural management type and (l**b**) carabid functional diversity andbiodiversity indices included in the systematic review (see Table 2 for abbreviations).

According to the systematic review, the first papers addressing the response of carabid beetles to agricultural management were published from 2000 to 2009, with no articles found in 2002. Since? then, the temporal trend of studies dealing with this topic has shown a steadily increasing rate, indicating that it is an emerging field of research. This was found when 55 articles were published between 2010 and 2019, respectively (Figure 2). Overall, the selected papers show that most of the studies were conducted mainly in developed countries, particularly in Germany (n = 19), France (n = 11), the United Kingdom (n = 7), and the United States (n = 5), while few studies were conduc ted in devetoping countries in Southern Africa (n = 6) and East Asia (n = 4), (Figure 3). In terms of agricultural management systems, conventional farming predominated in 28% of studies, followed by diversified farming systems (17%), integrated agricuature (16%) and semi-natural habitats (16%). Only a few studies were conducted on organic farming (13%) and conservation agriculture (10%) (Table 2; Figure 4a).

### 3.3 Carabid Response Variables to Agricultural Systems

We recorded five carabid biodiversity indices (41 studies: 25 positive, 8 negative, and 8 neutral) used to assess the effects of agricultural management type. Richness (13 stud-ies) and abundance (10 studies), followed by diversity (9 studies), were the three most frequently assessed indices. The next frequently used measure was community composition (i.e., the assemblage of the different species comprising the studied community), followed by evenness. Functional diversity was measured in only a small proportion of papers (28 studies: 19 positive, 7 negative, and 2 neutral) and mainly concerned trophic guilds (13 studies), body size (9 studies), dispersal ability, and wing type (6 studies) across agricultural management type (Table 2, Figure 4b).

## 4 Discussion

This review highlights the need to increase our knowledge of the use of carabid beetles as indicators of agroecosystem sustainability. The use of monitoring techniques such as indicator species to assess environmental change in agroecosystems is an emerging topicin research [43,44,47,48]. Because carabids clearly respond to agricultural management practices, they can play an important role in determining which practices in agroecosystems bring us closest to our goal of agroecosystem sustainability [36]. Despite the fact that the number of studies on carabids in agricultural systems is increasing globally, we have found that there is a clear geographical preponderance of studies from the most developed countries in Europe, with a large gap in the developing countries of Southern Africa and East Asia [49–52]. This implies that indicator species are prioritized in developed countries for monitoring the status of management practices in agroecosystems [53,54].

Carabid beetles are ubiquitous, stenotopic, and influenced by different agroecosystem management practices [55–58]. Conventional agricultural management has repeatedly been linked to negative effects on carabid diversity and functional traits [59–63]. The detrimental effects of this management can be explained by the fact that the synchronization of tillage timing and agrochemical application reduces carabid diversity by causing direct mortality and disturbance of overwintering sites [64–66]. This disturbance indirectly eliminates food sources and alters the habitat by changing the density, distribution, and composition of weeds [67,68]. According to Schröter et al. [65], these provide foliage and seeds to different species, control microclimate and soil moisture, and determine the degree of physical protection from predators and freedom of movement. For example, species that breed in autumn and overwinter as larvae in the soil are vulnerable to having their abundance affected by conventional tillage [69–71]. However, not all species decline due to such disturbance; certain species may be resistant or sensitive [72,73]. Studies conducted in conventional systems have shown that carabids can be used as indicators for monitoring ecological changes caused by management practices such as pesticide use and tillage methods [74–76].

Furthermore, our results show some preferences in using biodiversity indices, i.e., species richness and abundance as indicators of agricultural management conditions [77,78]. Species richness is one of the simplest measures of species diversity, and along with species abundance, it provides useful information about the state of different management practices [79]. However, there is a significant gap in the use of carabid functional diversity metrics. Trait-based information should also be included when assessing the sustainability of agroecosystems because measures of species richness and abundance are clearly insufficient to explain ecosystem functioning [50,52,80]. Dispersal ability, which is linked to functional traits such as body size, may be a key factor when assessing the health of agricultural management practices [81–84]. Body size has a greater influence on prey consumption [40]. This shows that functional traits, rather than broad community descriptors such as species richness, can better indicate ecosystem services of pest control for agricultural systems [43,50,85]. The dominance of key indicator species with high feeding rates can be used to assess the efficiency of a predator community in controlling pests [68]. For instance, larger carabids are a predictor of pest consumption, with larger species consuming large pests [21,47,63]. However, due to their limited dispersal ability, larger brachypterous predators are thought to be at greater risk of extinction than smaller macropterous species in severely disturbed and fragmented homogenized agroecosystems [57,73]. Smaller species thrive in open habitats because they can disperse when conditions are unfavorable [29,77,78], whereas species with restricted dispersal ability can only colonize new areas by running [36,56,86,87].

Carabid species that benefit from intact agroecosystems are considered indicators of sustainable management practices [88]. This implies that ecological, conservation, and integrated farming systems with a diverse crop mix and a diversity of field edges and strips are critical for the survival of carabid species [89,90]. Over time, these agroecosystems will provide shelter and more diverse food supplies, perhaps promoting carabid functioning and biodiversity [72,91]. Carabid species that occur in various habitats benefit from different management practices than specialists that occur in just one or a few habitats [92]. Each agroecosystem has its unique set of species, including both generalists and specialists, that can be used to track the effectiveness of management strategies [57,86].

## 5 Conclusions

Given the constraints associated with implementing sustainable measures in agroecosystems, we suggest that carabid beetles can aid in monitoring the recovery and health of agricultural management practices. Continuous monitoring of indicator species in agroecosystems can reveal the pace and direction of change within management practices [24,93]. Though carabids are receiving remarkably more attention as indicators of agroecosystem sustainability worldwide, there is still a need to quantify diversity or composition and the impact of species functioning on agroecosystems. Determining the functional diversity of carabid beetles in agroecosystems can help us better understand their ability to provide ecosystem services of pest control [94]. Therefore, selecting indices or descriptors that integrate functional diversity information is crucial for future sustainable agricultural efficiency.

## Supplementary Material

PRISM abstract checklist

PRISM checklist

Supplementary Material The following supporting information can be downloaded at https://www.mdpi.com/article/10.3390/su15053936/s1, Table S1: Studies included in the systematic review. Table S2: PRISMA 2020 for Abstracts Checklist; Table S3: PRISMA 2020 Checklist.

## Figures and Tables

**Table 1:** Categories used to classify studies found in the literature search.

**Category Items**	**Description**
Agricultural management	Studies on organic and diversified farming, conventional tillage, conservation, grazing practices, and grassland effects on carabid beetles
Field crops	Soybean, wheat, peas, maize, clover, sunflower, oats, barley, alfalfa, rice, millet, and sorghum
Biodiversity indices	Studies including diversity: abundance, richness, evenness, and composition
Functional guilds	The trophic level of carabids: predators/carnivores, omnivores, and granivores and their functions in agroecosystems
Functional traits	Studies that recorded dispersal ability and morphometrics

**Table 2:** Carabid functional diversity and biodiversity indices in different agricultural management types (CF-conventional farming; OF-organic farming; DFS-diversified farming system; CA-conservation agriculture; INT-integrated farming; SNH-semi-natural habitats).

Response Variables	Agricultural Management Type								
	Effect	CF	OF	DFS	CA	INT	SNH	Total	% Total
**Functional diversity**									
**Body size:** small, medium, and large	Positive	1	-	2	-	1	2	6	8.7
	Negative	1	-	1	-	-	-	2	2.9
	Neutral	-	-	-	-	1	-	1	1.4
**Trophic guilds:**									
predators, omnivores, and granivores	Positive	2	2	-	1	2	1	8	11.6
	Negative	1	-	1	-	-	2	4	5.8
	Neutral	1	-	-	-	-	-	1	1.4
**Dispersal ability** (wing type): flightless, immobile, macropterous, brachypterous, wingless, and apterous	Positive	1	1		2		1	5	7.2
	Negative	1	-	-	-	-	-	1	1.4
	Neutral	-	-	-	-	-	-	-	-
**Biodiversity indices**									
Richness	Positive	1	3	1	1	2	1	9	13.0
	Negative	1	-	1	-	-	-	2	2.9
	Neutral	1	-	-	-	-	1	2	2.9
Diversity	Positive	-	-	3	-	-	1	4	5.8
	Negative	2	-	1	-	1	-	4	5.8
	Neutral	1	-	-	-	-	-	1	1.4
Abundance/active density	Positive	2	1	1	2	1	-	7	10.1
	Negative	1	-	-	-	-	-	1	1.4
	Neutral	1	-	-	-	1	-	2	2.9
Composition	Positive	1	-	1	-	1	-	3	4.3
	Negative	1	-	-	-	-	-	1	1.4
	Neutral	-	-	1	-	-	1	2	2.9
Evenness	Positive	1	1	-	-	-	-	2	2.9
	Negative	-	-	-	-	-	-	-	0.0
	Neutral	-	-	-	1	-	-	1	1.4
Grand total		19	9	12	7	11	11	69	100

## Data Availability

Data can be obtained by contacting the corresponding author.
