# Supplementary material for: Carabid Beetles (Coleoptera) as Indicators of Sustainability in Agroecosystems: A Systematic Review
Source: Sustainability. Author manuscript; Available in PMC 2023 Sep 7. (PMC7615042; doi:10.3390/su15053936)
Supplement: Supplementary Material [file EMS187258-supplement-Supplementary_Material.pdf]

Table S1. Examples of documented studies on investigating the response of carabid beetle biodiversity and functional diversity to different agricultural management practices across continents and countries.

			Agricultural Management Type						
**Country/Reference**	**Factors investigated**	**Response variables**	**CF**	**OF**	**DFS**	**CA**	**INT**	**SNH**	**Reported comments**
Germany, [6]	Differently managed agroecosystems	Larger predators:<br>Less dispersal<br>Herbivore<br>Apterous	 <br><br>+	+ 					Organic management, as well as smaller fields and longer edges, increase carabid functional diversity.
Germany, [26]	Agricultural landscapes	Richness:<br>Macropterous	-	+					It is suggested that weedier and less densely cropped fields be tolerated to promote the agricultural carabid fauna.
USA, [29]	Diversified agroecosystems	Active density:<br>Granivorous	=		+				The importance of considering both direct and indirect effects of management in studies of invertebrate seed predators.
South Africa, [30]	Maize fields and adjacent natural vegetation under agricultural disturbance intensity	Diversity<br>Composition					-<br>-	+<br>+	Crop field margins of active cultivation are important conservation sites for the long-term survival of beneficial species and functional diversity arthropods
USA, [32]	Cover crop-based management	Active density<br>Small- Omnivores<br>Large carnivores<br>Granivores		 <br><br>+<br>+	 +<br><br>+<br>-				Management during the organic transition, including the importance of plant residue, reduced tillage, and the timing of cover crop termination dates determine carabid populations.
France, [34]	Differently managed systems	Active density:<br>Granivores<br>Carnivores<br>Omnivores	 -<br>-<br>+	 +<br>-<br>+					The abundance of seed-eating carabids has been found to be positively related to the level of seed predation.
Germany, [35]	Conversion from conventional to organic farming for 15 years	Richness		+					Organic farming support “open-habitat” species and increase their biodiversity.

South Africa, [40]	Agricultural managements	Diversity<br>Body size	-<br>-	+<br>+			Organic intercropped wheat agroecosystems support predatory carabid beetle body size and diversity.
England, [43]	Landscape structure and non-crop habitat	Active density:<br>Small Active<br>Large Flightless			 +<br>-	 +<br>+	Longer surveys in a variety of landscapes are likely to improve understanding of activity, diversity, and distribution of invertebrates, which are fundamental requirements if predators such as carabid beetles are required for ecosystem service provision.
UK, [44]	Environmental factors in agroecosystems	Abundance<br>Richness<br>Composition	 -<br>-	+ 			Diverse cropping and landscape heterogeneity at the farm scale can benefit individual species' preferences, help build diverse communities, and potentially increase service resilience and stability over time.
Germany, [47]	Disturbance in agroecosystems	Abundance<br>Richness	 -	+ 			Estimates of productivity and disturbance may have application in the provision of ecosystem services, particularly in determining the conditions required to optimize ground beetle activity for pest control.
UK, [48]	Contrasting conservation grazing management	Richness<br>Composition	- 	 +			The grazing regime has an impact on carabid beetle communities and is critical in maintaining distinct species compositions as well as rare and declining species.
Germany, [49]	Restored grassland	Dispersal ability<br>Trophic guilds			+<br><br>+		The preservation of well-connected woody seminatural habitats is critical for functionally diverse carabid assemblages with a high predator proportion.
England, [50]	Habitat type and landscape structure	Activity density<br>Richness<br>Macropterous<br>Dimorphic	 <br>- 	+<br>+<br><br>+			Predatory ground beetles rely more on landscape structure than phytophagous species.
Germany, [51]	Weed removal in agroecosystems	Activity-density<br>Richness	-<br>-	+<br>+			Organically managed fields increase carabid activity density and diversity and should be incorporated into future management strategies for natural enemy conservation.
Germany, [52]	Agri-Environment Schemes (AESs)	Dispersal ability:<br>Omnivorous<br>Herbivorous	=	=			Highlight the importance of local farming practices and edge habitats in maintaining the functional diversity of carabid assemblages in arable fields.

France, [54]	Management and landscape context	Abundance:				+			Local and landscape scales can be used to increase carabid beetle abundance and seed predation in arable fields.
		Carnivores				+			
		Omnivorous						+	
		Granivorous							
France, [55]	Different habitats in the farmland landscape	Activity-density			+				The beneficial role of oilseed rape in carabid species emergence from overwintering occurred primarily in the margins of oilseed rape-cropped fields.
France, [56]	Agri-Environment Schemes (AESs)	Activity-density	-	+					Non-targeted organisms can benefit from AES management.
		Richness	-	+					
France, [57]	Habitat type and landscape context of habitats	Abundances:					=		The spatial configuration and connectivity of annual crops in the landscape can contribute to an increase in the abundance of some carabid species.
		Brachypterous							
		Dimorphic					-		
		Macropterous					+		
Sweden, [59]	Field farming system	Richness	-	+					Local farming practices explain less variation in carabid responses than large-scale landscape context.
		Evenness	+	-					
Canada, [60]	Farming systems	Diversity		+	-				The annual-grain system was discovered to support more species diversity than the diverse grain-forage system.
Norway, [61]	6-8 years after the conversion from conventional to organic farming.	Activity-density	+	+					Even at the species level, biological farming has a positive impact on carabids.
		Diversity	-	=					
Germany, [62]	Agricultural landscape	Dispersal ability		+					Grassy strips have no effect on the dispersal of typical arable field species; however, species that require grass vegetation can only overcome arable field barriers by using grassy strips alone or in conjunction with an adjacent hedge.
Zambia, [63]	Agricultural managements	Activity density	-						Carabid assemblage size structure is influenced by disturbances as well as competitive interactions.
		Body length:							
		Smallest Largest	-						
Australia, [64]	Contrasting farmland uses in a mixed-farming landscape	Morphological							Farmland management has an impact on body size and dispersal-related traits in farmland and adjacent native vegetation.
		traits: Smaller			+				
		Larger							
		Wingless			+				

		Reduced wings			=				
Germany, [65]	10 years after the conversion from conventional to organic farming	Abundance<br>Richness	+<br>+	+<br>+					Species benefited by organic farming were abundant outside arable fields during the conventional period.
Switzerland, [66]	Different low-input farming systems	Abundance	-	+					Seminatural habitats in conjunction with organic farming may be an important factor in the conservation and enhancement of species rich assemblages on agricultural land.
Germany, [67]	Agri-Environment Schemes (AESs)	Active density:<br>Predatory<br>Granivorous<br>Richness	 -<br>+<br>-				 +<br><br>+		Beneficial effect of adjacent agri-environmental schemes (AES) on ground- dwelling predators in oilseed rape (OSR).
France, [68]	Management of field margins	Active density:<br>Large<br>Smaller<br>Medium	+<br>-<br>+<br>-				+	+<br>+	Diversity in carabid responses to the vegetation structure appears to be related not only to variation in their body size, but also in other life history traits such as diet.
China, [70]	Variations in environmental conditions at local landscape	Active density<br>Richness<br>Composition	-<br>-		+<br>+<br>+				Provide critical insights for developing more effective pest-suppression intercropping systems.
China, [71]	Plant diversity, habitat type and landscape structure	Diversity:<br>Small-omnivorous<br>Composition:<br>Large-predatory						 =<br><br>=	The preservation of extensively managed habitats, in conjunction with a targeted increase in local plant diversity, is critical for optimizing biological pest control by carabid assemblages.
France, [73]	Spatial and multi-year temporal heterogeneity of the crop mosaic	Abundance:<br>Dispersal power<br>Less mobile			+				The significance of taking crop heterogeneity into account in future ecological studies on biodiversity in agricultural landscapes.
Zimbabwe, [74]	Tillage system fertilizer application rate and weeding intensity	Diversity:<br>Detritivores<br>Herbivores	 -<br>-			 +<br>+			Conservation tillage with crop residue retention has the potential to increase the density and diversity of beneficial beetle species.

UK, [75]	Crop type and Landscape-scale	Abundance:<br>Predators<br>phytophagous	 -<br>-				The presence of grassy field margins in arable landscapes increases carabid abundance.
Germany, [76]	Agricultural management and landscape context	Active density<br>Richness	-<br>-	+<br>+			Ground arthropod diversity and the associated ecosystem functions may benefit from increased environmental heterogeneity in organic management.
Europe, [77]	Small- vs. large-scale agricultural landscapes	Diversity			+	+	Semi-natural landscape features, such as agroforestry tree components, can contribute to functional agrobiodiversity.
Italy, [78]	Cutting regime of grass on rice field banks	Abundance<br>Diversity<br>Functional composition:<br>Generalist<br>Predatory-short-winged				=<br>+<br><br>+<br>-	Environmentally friendly managed banks may aid in the survival of species with poor dispersal ability and predator populations.
Canada, [79]	Landscape context	Active density<br>Diversity<br>Composition	+<br>=<br>-		-<br>+<br>+		Intercrops of canola and wheat have the potential to increase populations of some carabid species, putting additional pressure on some canola insect pests.
USA, [80]	Tillage methods	Active density<br>Diversity	+<br>-	-<br>+			Some species were affected directly by tillage, while others were affected indirectly by habitat characteristics. CT may provide habitat that xerophilic spring breeders prefer.
Canada, [81]	Agronomic practices and the local environment, landscape structure	Abundance<br>Diversity			+<br>-	+<br>+	Environmental management should consider not only the local characteristics of the habitat but also the characteristics of the surrounding landscape to increase predatory beetle abundance and diversity.
USA, [82]	Restored grassland	Diversity<br>Composition<br>Small-macropterous-phytophagous<br>Large-Flightless-Carnivores	-<br>-<br><br>-		+<br>+<br>+<br><br>+	 <br>+<br>+	To determine the functional importance of ground beetle community re-establishment, measurements of actual functions and interactions, such as seed and arthropod predation, will be required.

France, [83]	Landscape context during the last five years	Richness<br>Composition				+<br>+	+<br>-	Old semi-natural field margins should not be regarded as undisturbed habitats, but rather as management units in agricultural landscapes, such as arable fields.
Europe, [84]	Non-crop habitats	Active density<br>Richness	-				+	Non-crop habitat could enhance the diversity of ground-dwelling carabids in agricultural landscapes
England, [85]	Differently managed systems	Abundance<br>Diversity	=	+<br>-				Lower carabid diversity in organic farms is due to a significant increase in the dominance of a single species.
Scotland, [86]	Agricultural management	Composition: Predators	-				+	Habitat classification may help in predicting how such changes to the community structure may influence ecosystem functioning.
Italy, [87]	Management regimes	Richness<br>Short winged, large predatory	 -				 +	Ecological traits must be standardized and incorporated in the assessment and monitoring of the impact of human activities on the environment.
France, [88]	Spatio-temporal heterogeneity	Active density<br>Richness				-<br>=	+<br>-	Adjacencies between woody and cultivated habitats play a role in the conservation of a diverse carabid assemblage in winter cereals and maize.
Sweden, [89]	Landscape, habitat, and farm management	Composition	-	+		+	+	Diverse biodiversity conservation strategies in agroecosystems are required to increase viable populations of various groups of organisms including carabid beetles
Canada, [90]	Field density and distribution of weeds	Activity-density: omnivorous	=					Presence of weedy patches of vegetation in cropped areas contributes to biodiversity by conserving populations of carabids, which also increases rates of weed seed predation.
Finland, [91]	Landscape-scale and proportions of leys and semi-natural grasslands	Diversity: large	+	+				Arable weed diversity is affected by organic farming to a higher extent than carabids.
USA, [92]	Farm management strategies	Activity–density<br>Richness			+<br>+			Both grazing and mowing serve as ecological filters for weed and carabid beetle communities.

Germany, [93]	Landscape complexity	Active density	-	+					Landscape features were much more important than organic farming management for enhancement of local biodiversity and should thus be considered in Agri-environment schemes.
		Richness	=	=					
Sweden, [94]	Agricultural land use	Abundance							Diverse mix of agricultural land uses will provide high levels of predators from different functional groups.
		Macropterous-omnivorous	+						
		Brachypterous-predators						+	

*CF- includes the use of agroechemicals, intensive tillage and, monoculture production, ; OF- No pesticides application, uses fertilizers such as compost manure, green manure; DFS-multiple crop planting in the same field, ecological diversity at plot, field, and landscape scales; CA-promotes minimum soil disturbance (i.e. no tillage), maintenance of permanent soil cover, and crop rotation; INT-integrate livestock and crop production; SNH- not subjected to intense farming, such as an area of field margins,(i.e., flower strips, hedgerow). The overall agroecosystem management effect on carabids, as reported by the study, is also recorded, and noted as positive response (+), neutral response (=), negative response (-).
